# Two Sides of the Same Coin: The Intersection Between Psychiatry and Acute Care

**DOI:** 10.1192/bjo.2026.11787

**Published:** 2026-06-30

**Authors:** Naduni Jayasinghe

**Affiliations:** Prospect Park Hospital, Reading, United Kingdom

## Abstract

**Aims:**

Psychiatric inpatient units are often geographically, organisationally and culturally distinct from tertiary care hospitals, creating separation. When these two worlds inevitably collide, challenges often arise. Evidently, there is a limited understanding of each team’s challenges and, in this game of tug-of-war, patients bear the ultimate price.

**Methods:**

**Case Presentation**

A 67-year-old man with dementia was admitted to a psychiatric inpatient unit for management of behavioural and psychological symptoms. During admission, he developed an acute change in mental state, consistent with delirium. He was therefore referred to tertiary care, where investigations revealed urinary retention, with an estimated three litres in the bladder.

Despite discussions between the emergency department (ED) and Psychiatry, the patient was discharged back to the psychiatry unit, with advice for catheterisation and intravenous (IV) antibiotics. Unfortunately, mental health wards could not perform these tasks due to a lack of experience, staff and equipment. Hence, the patient was transferred back to ED. On re-presentation, twenty-four hours after initial escalation, the patient became unconscious, was intubated and admitted to intensive care.

**Results:**

This case illustrates the systemic challenges posed by the mental-physical health interface. From a psychiatric perspective, there was a sense of frustration and helplessness, whilst ED was similarly challenged due to the complexity of the patient’s confusion. Pressures on services to remain efficient and meet targets, coupled with the disconnect between psychiatry and acute care, led to poor understanding of each party’s limitations, meaning the underlying cause of the patient’s life-threatening distress remained untreated. Limitations include a psychiatric unit’s inability to administer IV medications, lack of medically trained psychiatry staff and the increasing demands placed on services. Inadequate awareness of these challenges, along with the patient’s dementia, legal status, and psychiatric admission, may have subtly influenced how their needs were prioritised.

**Conclusion:**

The structural separation between mental health services and acute medical care within the UK healthcare system continues to widen, despite ongoing efforts from psychiatry liaison services. This case underscores the need for a shared understanding of resources, capabilities and clinical responsibility across services. Fragmentation between teams can lead to ambiguity, with acute medical services overestimating the capabilities of psychiatric units due to their designation as tertiary hospitals, while mental health services lack the infrastructure to deliver routine medical care.

Improved education, cross-service exposure and enhanced ward provisions may strengthen communication, clarify escalation pathways and, ultimately, improve patient outcomes.

